# A prospective stepped wedge cohort evaluation of the new national trauma team activation criteria in Sweden – the TRAUMALERT study

**DOI:** 10.1186/s13049-019-0619-1

**Published:** 2019-04-30

**Authors:** Fredrik Linder, Lina Holmberg, Martin Bjorck, Claes Juhlin, Knut Thorbjornsen, Jan Wisinger, Per Polleryd, Hampus Eklof, Kevin Mani

**Affiliations:** 1 Department of Surgical Sciences, Uppsala University, 75185 Uppsala, Sweden; 2 Department of surgery, Gävle county hospital, Gävle, Sweden; 3 Department of surgery, Västerås county hospital, Västerås, Sweden; 4 Department of surgery, Karlstad county hospital, Karlstad, Sweden

**Keywords:** Wounds and injuries, Trauma, Triage, Patient safety, Prospective stepped wedge cohort design, Epidemiology

## Abstract

**Background:**

Trauma triage based on prehospital information facilitates correct allocation of in-hospital resources. The Swedish national two-tier trauma team activation (TTA) criteria were revised in 2016. The current study aimed to evaluate the safety and efficacy of the new criteria.

**Methods:**

Five centres covering trauma care for 1.2 million inhabitants registered all trauma patients prospectively in the Swedish trauma registry (SweTrau) prior to and after stepwise introduction of new TTA criteria within the cohort (a prospective stepped-wedge cohort study design; period August 2016–November 2017). Evaluation of full- and limited-TTA frequency, under- and overtriage were performed at equal duration before and after this change.

**Results:**

The centres registered 1948 patients, 1882 (96.6%) of which were included in the study. With new criteria, frequency of full-TTA was unchanged, while limited-TTA decreased with 46.3% (from 988 to 531). 30-day trauma mortality was unchanged. The overtriage was 107/150 (71.3%) with former criteria, and 104/144 (72.2%) with new criteria, *p* = 0.866. Undertriage was 50/1037 (4.8%) versus 39/551 (7.1%), *p* = 0.063. Undertriage was consistently > 20% in patients with fall injury. Among patients with Injury Severity Score (ISS) > 15, 50/93 (53.8%) did not initiate full-TTA with former, vs 39/79 (49.4%) with new criteria, *p* = 0.565. Age > 60-years was a risk factor for undertriage (OR 2.89, *p* < 0.001), while low fall injuries indicated a trend (OR 2.70, *p* = 0.051).

**Conclusions:**

The newly implemented Swedish TTA criteria result in a reduction in limited TTA frequency, indicating an increased efficiency in use of resources. The over- and undertriage is unchanged compared to former criteria, thus upholding patient safety.

**Electronic supplementary material:**

The online version of this article (10.1186/s13049-019-0619-1) contains supplementary material, which is available to authorized users.

## Background

In trauma care, a multidisciplinary, resource intensive acute trauma patient management is crucial to maximally utilize the “golden hour” [1] during which effective resuscitative interventions are performed [2]. This acute trauma care is initiated through activation of a trauma team which often consists of multiple medical specialties, support staff and resource allocation with operating capabilities and CT-scanner availability.

Whilst rapid trauma team activation (TTA) is a cornerstone in trauma care, inadequate TTA may drain resources from other patients. Therefore, triage is an important tool to direct medical efforts towards patients with the most urgent needs [3, 4]. The American College of Surgeons Committee on Trauma (ACS-CoT) and the Center for Disease Control and Prevention (CDC) have published Guidelines for Field Triage of injured patients [4]. A two-tier trauma alert system is often used. Physiologic derangement or specified anatomical injuries in trauma patients initiate a full TTA, whilst Mechanism of Injury (MOI) criteria alone initiate a limited trauma alert [5, 6]. The efficacy of trauma triage criteria is evaluated with assessment of over- and undertriage, (Additional file 1: Table S1) [7].

The perfect triage system is assumed to activate a full TTA for all severely injured patients, and a limited or no TTA for patients with minor or no injuries. The Injury Severity Score (ISS) is a method to define the severity of injury during trauma. A patient with ISS > 15 is regarded as severely injured, and should thus initiate a full TTA when using the optimal triage system. The ISS is calculated using the Abbreviated Injury Score (AIS) where injures in different body areas are scored. The ISS is used as basis for evaluation of over- and undertriage. Overtriage is an assessment of what proportion of full TTAs that are activated by patients with minor injuries (ISS ≤15), i.e. resulting in unnecessary use of hospital resources. Undertriage, on the other hand, assesses the proportion of of severely injured patients with ISS > 15 not intiating full TTA. Over- and undertriage are the primary markers of how successful a triage system is in allocating adequate resources to patients in real life.

The development of effective triage criteria is a challenge [8–10]. In Sweden, modified two-tier ACS-CoT trauma triage criteria have been used, Table 1. Evaluation of these criteria in a retrospective cohort indicates that although they do result in an acceptable over- and undertriage, they also result in many limited trauma team activations in un-injured patients [11]. In 2015, the Swedish trauma association created a multidisciplinary workgroup involving twenty professional trauma-related organizations, with the task to develop national consensus-based TTA guidelines. The new TTA algorithm (Table 1) was introduced in late 2016 and implemented nationally in a step-wise fashion.

**Table 1:**
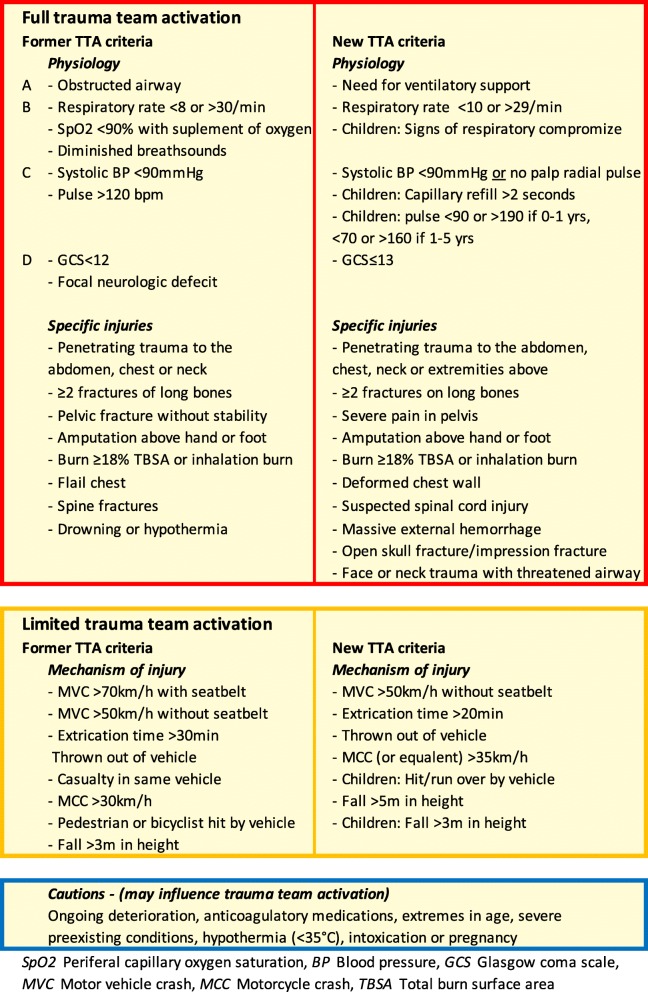
Former and new trauma team activation criteria

The aim of this study was to evaluate the safety and efficacy of the new national TTA criteria in terms of over- and undertriage compared to the previously used algorithm.

## Methods

### Study design

The study was performed in a population based setting in five Swedish hospitals covering trauma care for a total population of 1.2 million inhabitants. The actual change in TTA criteria was planned for implementation March 1st 2017 all over Sweden, and the study was planned and initiated in the spring of 2016. Initially the plan was a prospective before- and after cohort study but as is often the case with simultaneous clinical guideline implementations at different institutions, the actual change in TTA criteria took place at different times at the including hospitals. The study design was thus adapted to a stepped wedge cluster design of the introduction of the new trauma triage criteria. The evaluation of the TTA criteria in this cohort was performed using the prospective registration of trauma patients in the Swedish trauma registry (SweTrau). A pre-study power calculation indicated that to detect a change in undertriage from 4 to 8% with the Matrix method [7] with 80% power and 5% significance, a sample size of 553 patients per group was required.

### Swedish trauma registry and participating hospitals

SweTrau is a national trauma registry in Sweden established in 2011, based on “the revised Utstein Trauma Template for Uniform Reporting of Data following Major Trauma, 2009” [12]. The registry includes all patients where a full or limited trauma alert has been activated, as well as all trauma patients with a New Injury Severity Score (NISS) > 15. This also includes trauma patients secondarily transported to a higher-level trauma centre. Forty-eight hospitals, including all but one university hospital, participate in the registry [13].

**Fig. 1:**
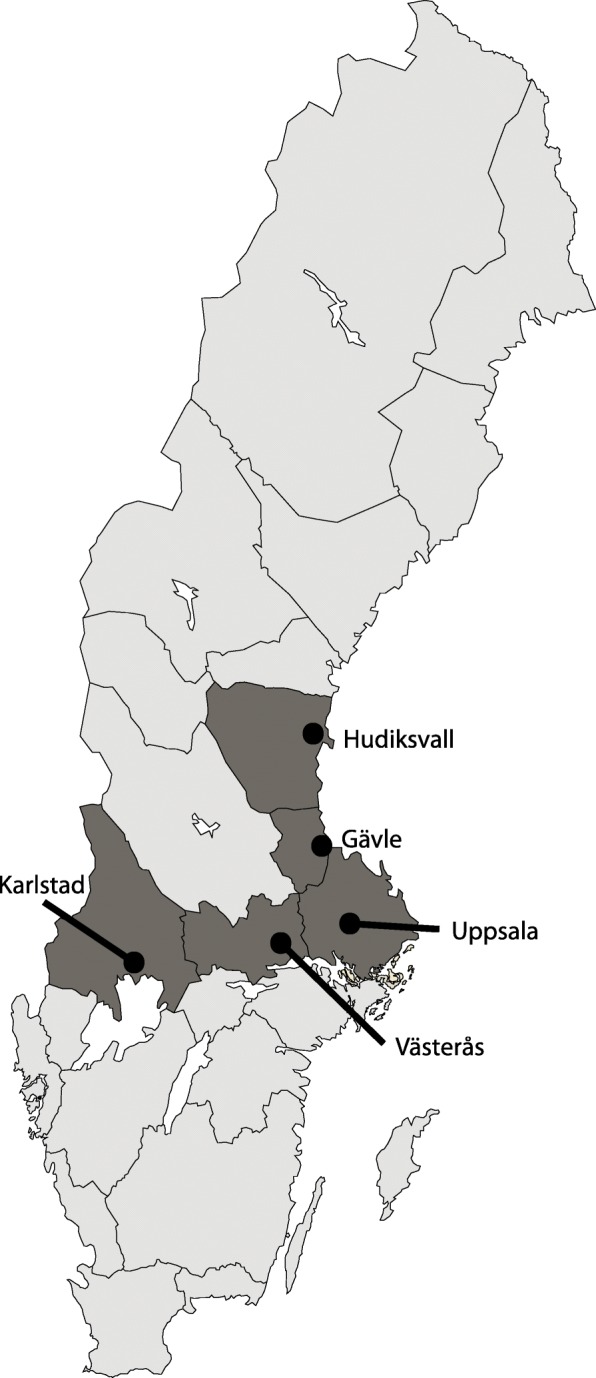
Map of Sweden presenting the geographical area that is covered for trauma by the five hospitals participating in this study

**Fig. 2:**
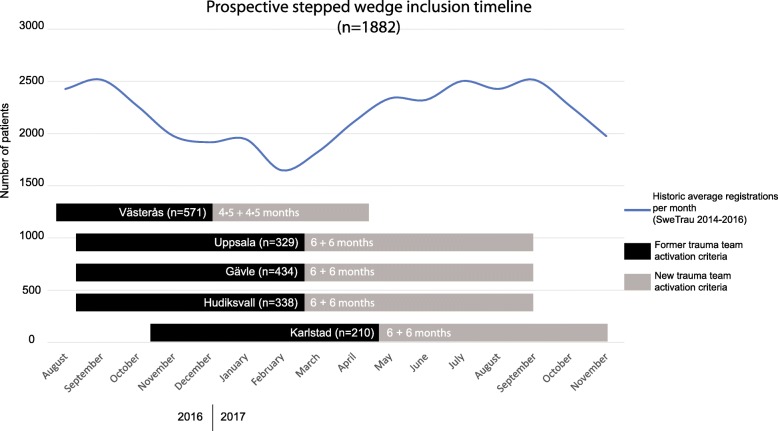
Illustration of the step-wedge prospective inclusion of patients in the study, including the time for change of Trauma Team Activation (TTA) criteria, per center. The number of trauma cases registered in the Swedish trauma registry per month during the period 2014–2016 is illustrated for reference

Five hospitals in the mid-Sweden participated in the current study, Fig. 1. These hospitals had harmonized criteria for full- and limited TTA prior to the transition to the new national criteria, which were stepwise implemented in the region, Fig. 2. All primary trauma patients registered in SweTrau at these centres before and after the change of TTA criteria were included in the study. Patients registered after secondary trauma transfer were excluded, as well as patients where information on TTA level or injury severity was lacking. The evaluation period was 2 × 6 months in four and 2 × 4.5 months in one hospital, Fig. 2.

### Triage

In-hospital triage was performed by a senior nurse according to guidelines when contacted by the pre-hospital staff, or by a senior anaesthesiologist in the medical helicopter service. At one of the hospitals (Västerås county hospital), the triage was performed by prehospital staff and reported by phone to the receiving nurse prior to the change in TTA criteria. After implementation of the new criteria, all hospitals performed in-hospital triage by a senior nurse. The effect of this practice change on outcome was assessed in a sensitivity analyses (see below).

### The trauma team

The resources allocated differ between limited and full TTA. The initial survey is conducted by the surgeon on call for limited TTA, and as a team effort for full TTA. A senior consultant specialized in trauma care receives a “stand by call” during limited TTA if the alert is upgraded and this consultant is designated trauma leader for full TTA. The affiliated staff for full, limited and no TTA is listed in Table 2.

**Table 2:** Study population demographics after subdivision into the studied groups

	Former criteria (*n* = 1187)	New criteria (*n* = 695)	*p*-value
Age – years, median (IQR):	36 (21–60)	40 (22–61)	0.173
Full trauma team activation	40.5 (23–63)	43 (22–62)	0.726
Limited or no trauma team activation	36 (21–59)	40 (21–61)	0.129
Male gender, % (n):	59.0 (704)	64.6 (450)	0.013
Penetrating trauma, %(n):	2.4 (29)	7.8 (54)	< 0.001
Glasgow Coma Scale (GCS) in Emergency			
Department, % (n):			
Normal or minimal injury (GCS 15)	84.4 (1037)	82.3 (572)	0.003
Mild injury (GCS 13–14)	5.1 (60)	8.6 (60)	0.002
Moderate injury (GCS 9–12)	1.1 (13)	3.6 (25)	< 0.001
Severe injury (GCS 8 or below)	2.1 (25)	2.9 (20)	0.290
ASA score, median (IQR):	1 (1–2)	1 (1–2)	0.254
Injury Severity Score, median (IQR):			
Full trauma team activation	8 (1–16)	9 (1–16)	0.977
Limited or no trauma team activation	1 (0–4)	2 (1–8)	< 0.001
New Injury Severity Score (NISS), median (IQR):			
Full trauma team activation	9 (3–25)	9 (2–21)	0.848
Limited or no trauma team activation	2 (0–4)	3 (1–9)	< 0.001
30-day mortality % (n):			
Full trauma team activation	13.8 (20)	12.1 (17)	0.662
Limited or no trauma team activation	1.7 (18)	2.6 (14)	0.268

ASA: American Society of Anesthesiologists physical status classification; IQR: Inter-quartile range

### Evaluation of safety and efficacy

ISS was used as basis for evaluation of over- and undertriage. The choice of ISS, rather than NISS (which serves as inclusion for SweTrau) for this evaluation was based on the fact that NISS is regarded as less accurate in predicting risk of mortality in blunt trauma [14]. Additionally, the ACS-CoT recommendations for acceptable levels of over- and undertriage are based on ISS^7^. The ISS was calculated for all patients using the Abbreviated Injury Score 2005 rev 2008 (AIS). The actual scoring was performed by accredited AIS-scoring professionals at all including hospitals using the AIS-module of the trauma registry. The most severe cases where validated by re-scoring at another including hospital to ensure accuracy and adequacy in the scoring. Patients with an ISS > 15 were considered severely injured [15, 16]. Over- and undertriage was calculated using the Matrix method, Additional file 1: Table S1 [7]. Additionally, the proportion of severely injured patients who did not initiate full TTA was evaluated with former and new criteria as a measure of safety, (Additional file 1: Table S1) [17]. This additional method was necessary as the design of the new criteria aimed to reduce the number of limited trauma alerts, hence per se influencing the denominator in the evaluation of undertriage based on the Matrix method. Changes in number of TTA were evaluated in four different groups based on ISS (0–15, 16–24, 25–49 vs 50–75) [18, 19].

### Sensitivity analyses

To evaluate the potential effect of the differing triage routine in Västerås county hospital on overall outcome, TTA frequency and over- and undertriage were calculated separately when excluding this hospital in a sensitivity analysis.

As the implementation of new TTA criteria occurred late 2016/early 2017 in most hospitals, the former criteria were primarily used during autumn and winter months, whilst the new criteria were more often used during spring and summer months. To assess the potential effect of the seasonal variation on TTA frequency, an evaluation of the number of TTAs registered in the SweTrau during different months for the period 2014–2016 was performed in relation to the current study.

### Statistics

Data were assessed for normality with histograms. Categorical data were reported as ratios with 95% confidence intervals (CI), and were assessed with chi-square. Non-normally distributed data were reported as medians with interquartile range (IQR) and compared with Mann-Whitney-U test. Predictors for undertriage were assessed in a multivariable binary logistic regression analysis. A *p*-value of < 0.05 was regarded as significant. Statistical analyses were performed with IBM SPSS Statistics version 25 (IBM Corp, Armonk, New York, USA).

## Results

**Fig. 3:**
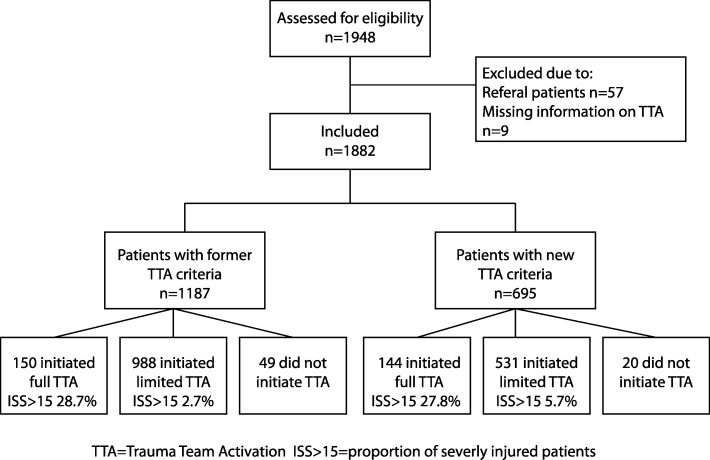
Flow chart of patients included in the study

During the study period, 1948 trauma patients were registered in SweTrau at the participating centres, 66 (3.4%) of which were excluded, Fig. 3. The numbers of patients initiating full TTA, limited TTA and no TTA before and after change of trauma criteria are presented in the flow chart, Fig. 3. The number of patients receiving full TTA and those not initiating TTA was not significantly different in the two cohorts (before and after implementation of the new criteria). There was a 46.3% (95% CI 43.1–49.4%) decline in the number of cases leading to a limited TTA in the cohort in which the new criteria was implemented (988 limited TTA with former criteria vs 531 limited TTA with new criteria, Fig. 3.

Study population demographics are presented in Table 2. The proportion of male patients activating any alert increased with the new criteria, as did the median ISS of patients with limited TTA. There was no difference in 30-day mortality after trauma based on the triage criteria.

**Fig. 4:**
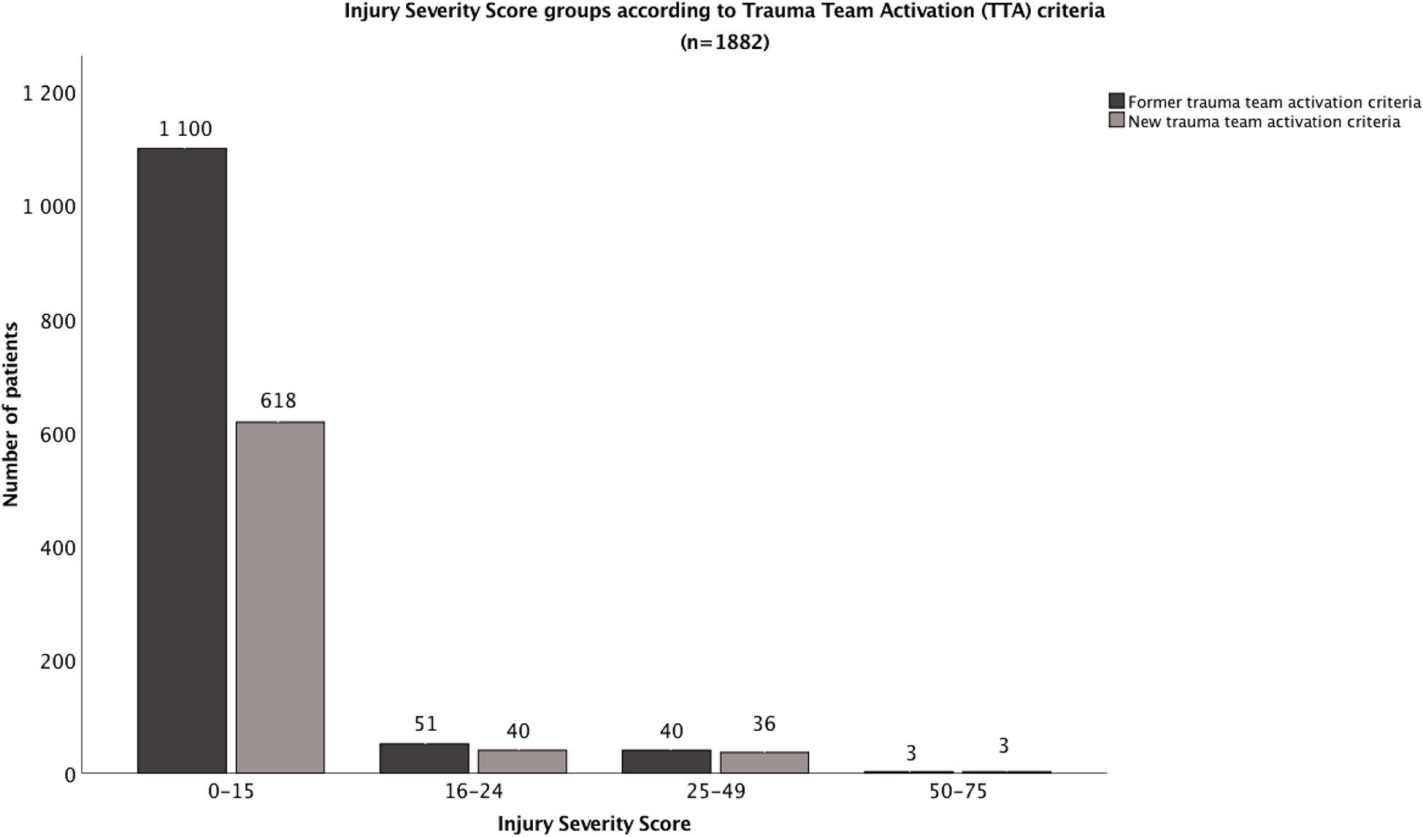
Number of trauma team activations (TTA) with former and new criteria according to injury severity score (ISS) group

To assess if reduction in TTA activation occurred in patients with severe injury or not, all patients were divided into four subgroups according to their ISS values [19] as presented in Fig. 4. The most important reduction in number of limited TTAs occurred in the patients that were not severely injured (ISS 0–15, decrease in TTA by 48.1% with new compared to former criteria). Overall, 99.1% of the reduction occurred in patients with ISS ≤ 15, and 87.8% of the reduction was in the group of patients with ISS 0–2.

### Over- and undertriage

Overtriage was 71.3% (107/150 patients) with the former TTA criteria and 72.2% (104/144 patients) with the new TTA criteria, *p* = 0.866. Undertriage calculated with the Matrix method was 4.8% (50/1037) with former criteria and 7.1% (39/551) with new criteria, *p* = 0.063. When assessing the severely injured patients (ISS > 15), 53.8% (50/93) did not initiate full-TTA with former, vs 49.4% (39/79) with new criteria, *p* = 0.565.

### Subgroup analysis of undertriage

**Fig. 5:**
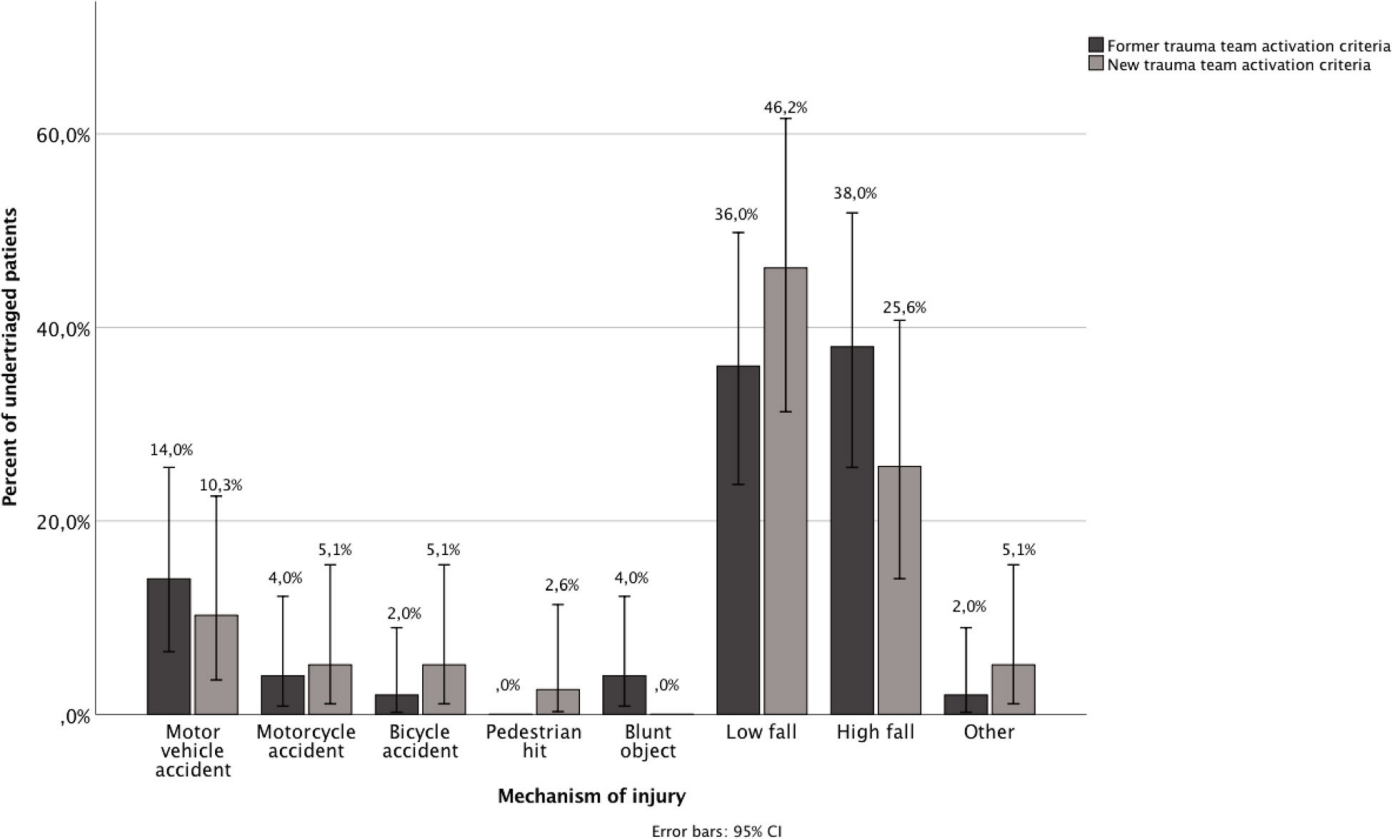
Proportion of undertriaged patients based on mechanism of injury. Error bars indicate 95% confidence interval

Undertriaged patients according to injury mechanism are presented in Fig. 5. Road traffic accidents constitute 55.3% (656/1187) of all patients with former criteria and 52.9% (368/695) with new criteria. Ten patients subject to road traffic accidents were undertriaged with former and nine with new criteria, *p* = 0.204. Patients subject to low fall injury constituted 13.8% of the trauma patients (164/1187) with former and 12.2% (85/695) with new criteria, *p* = 0.327. Eighteen of these patients were undertriaged with both former and new criteria *p* = 0.051.

Binary logistic regression analyses of risk factors for undertriage indicate a higher risk for undertriage in patients ≥60 years of age, Table 3. There was a trend for increased risk for undertriage in patients with fall injury.

**Table 3:** Odds-ratio of risk factors for undertriage

	Odds-ratio	95% CI	*p*-value
Mechanism of injury			
Motor vehicle accident	0.431	0.15–1.27	0.126
Motorcycle accident	0.743	0.19–2.84	0.664
Bicycle accident	0.546	0.13–2.35	0.416
Pedestrian hit	0.563	0.06–4.99	0.606
Gunshot wound	0.000	0.00 -	1.000
Stab wound	0.000	0.00 -	1.000
Low fall injury	2.704	1.00–7.35	0.051
High fall injury	1.681	0.63–4.47	0.298
Age ≥ 60 years	2.886	1.74–4.79	< 0.001
ASA score ≥ 3	0.935	0.53–1.66	0.818

ASA: American Society of Anesthesiologists physical status classification

### Sensitivity analyses

When excluding Västerås county hospital, 1311 patients remained for analysis. The number of limited TTA decreased from 707 to 316 after change of TTA criteria (55.3% reduction, *p* = < 0.001). The number of full TTA as well as the number of patients not initiating TTA was unchanged. Overtriage was 67.5% with former criteria, versus 68.2% with new, *p* = 0.915. Undertriage with Matrix method was 5.9% versus 7.6%, *p* = 0.281. Among severely injured patients, 53.7% did not initiate full TTA with former criteria, versus 67.6% with new criteria, *p* = 0.168. Thus, overall the sensitivity analysis indicates the same trend as in the overall cohort, with reduction of limited TTA and stable over- and undertriage.

The re-scoring of AIS in severe cases between hospitals did result in minor variances regarding AIS-codes for injuries but it did not result in any differences regarding AIS-score and subsequently no differences in ISS for any patient.

In order to analyse the seasonal variation in TTA, the SweTrau registry was consulted for the period 2014–2016. The mean number of TTA registered in SweTrau during the months of September–December, which was the period during which the former criteria were used at most centres, was 2889. During the period May–August (new criteria), the number of registered cases was in average 3196. The monthly national trend in TTA registration in SweTrau is presented in Fig. 2.

## Discussion

The current analysis confirms that the updated national TTA criteria in Sweden are safe, with levels of over- and undertriage remaining unchanged compared to former criteria. Additionally, the new criteria put less strain on acute care in-hospital resources. They result in a significant decrease in the number of limited TTA, without compromising patient safety. However, the analysis of undertriage in the current paper indicates that further modification of the TTA criteria may be required for specific patient cohorts and MOI. This concerns particularly elderly patients and those subject to fall injury, where undertriage was higher than expected.

In this study, a prospective stepped wedge cohort design was used to evaluate change in triage criteria. The optimal methodology to study such intervention without bias would have been a randomized controlled study, where patients would have been triaged with one of the two criteria based on randomization. Although we initially aimed for such a study design, it was not accepted by the ethical review board. They required informed consent prior to randomization from all patients, which we regarded as impossible to achieve. Unfortunately, Swedish law does not accept randomization without informed consent, in contrast to for instance the UK, where the Mental Capacity Act makes a two stage consent process possible [20]. The prospective stepped wedge cohort design is a pragmatic and suitable choice for studying an intervention on a system level [21], sometimes called a pseudo-randomization. The benefits of such study design include the fact that the introduction of a new routine resembles a cluster randomization, controlling for bias. The stepped wedge introduction of the intervention to some extent controls for changes due to temporal trends. The population-based element of this study, and the use of an established trauma registry [13], increases the generalizability of the analysis.

The main change in the new Swedish TTA criteria compared to the former ones is the substantial reduction in MOI criteria for initiating limited TTA. This is based on several studies questioning the validity of MOI criteria in predicting severe injury [5, 22, 23]. The MOI remaining in the new criteria are either evidence based (fall≥5 m, extrication time > 20 min) [5] or of a more obvious nature (thrown out of vehicle, child struck by car) where the consensus group could not agree upon their removal. The important reduction in limited TTA with the new criteria (which almost exclusively occurred in patients with ISS ≤ 15) in combination with maintained undertriage level supports the consensus group’s aim in judiciously restraining limited TTA criteria without jeopardizing patient safety.

The power-estimation was performed with the hypothesis that if undertriage according to the Matrix method increased from 4 to 8%, it would be reliably detected. The method of choice for evaluation of undertriage is a matter of debate, as the Matrix method includes an inherent error, where undertriage is affected by the total number of patients initiating a limited or no TTA. In the current study, the change of TTA criteria per se resulted in a reduction in number of patients initiating a limited TTA, and thus reduced the denominator for the calculation of undertriage according to the Matrix method, Additional file 1: Table S1 [17]. This resulted in an increase in undertriage calculated with this method (from 4.8 to 7.1%). Although this increase was not statistically significant, this could be due to a type II error. Additionally, based on the Matrix method, the undertriage for the new criteria increased to above the recommended level of 5% based on ACS-CoT recommendations.

The method of calculating undertriage based on proportion of severely injured patients not initiating a TTA is more robust when assessing triage over time regardless of TTA criteria [17]. The main weakness of the method is that there is no current definition on what is an acceptable percentage for undertriage. In our study, the undertriage was close to 50% with this method. Although the overall rate of undertriage calculated with this method was unchanged when comparing the former and new criteria, the fact that approximately half of the patients with severe injury did not initiate a full TTA indicates that there may be a need for further revision of the criteria. A detailed analysis of the trauma mechanism and injury panorama in these patients would inform this process. An important aspect is to assess compliance to TTA criteria, i.e. that all patients who meet the criteria also receive TTA. A previous study indicates that compliance may affect both the cost-efficiency of TTA as well as patient safety [11].

The results of the current study highlights a persistent problem: the inability of former as well as new TTA criteria to successfully reduce undertriage in elderly patients and those subject to injury from fall. Many of these severely injured patients who are undertriaged have single life-threatening injuries, however, and may not always benefit from a full trauma team. Future studies with the aim to reduce undertriage in these patients, or to improve the resource allocation in an alternative way, are warranted.

The overtriage rate was unchanged during the study period (71–72%), and was substantially higher than the 35–50% which is recommended by the ACS-CoT. This indicates a potential for further sharpening of the triage criteria to reduce remaining inefficiencies. During the TTA criteria revision, pulse rate was excluded as a TTA criteria because of the lack of evidence for its validity as a standalone criterion [24]. The shock index (SI, ie the ratio of heart rate to systolic blood pressure) has been proven in several studies to accurately predict severe injury [25, 26], and could be considered for future revisions of the criteria.

### Limitations

The prospective stepped wedge design of this study does not compare TTA during the same time of year while both mechanisms of trauma and prevalence is seasonal. An analysis of trauma registration in the SweTrau showed that the number of trauma in Sweden generally is higher during the summer months, when the new criteria were evaluated in the current cohort, Fig. 2. The reduction in the number of TTA with the new criteria despite evaluation during the summer months indicates that the effect of the new criteria in lowering the number of TTA may even have been underestimated.

While the data was analysed regarding undertriage for specific mechanisms of injury, the study was not powered for the subgroup analyses, introducing a risk for type II error. Additionally, there was no information available regarding the impact of each specific TTA criteria in triggering each TTA level. A future prospective study assessing the efficacy of each specific criteria for full TTA could amend current evidence and assist in further revision of TTA criteria.

The adequacy of triage criteria is affected by the trauma panorama and the prevalence of MOI in the studied population. The population-based setting of this study ensures that the analysis is relevant for a Scandinavian/Western European population, e.g. where blunt trauma mechanism is more prevalent than penetrating trauma, and where the vehicles involved in road traffic accidents are relatively modern with adequate safety equipment. The under- and overtriage with these criteria may vary in other populations.

## Conclusions

The newly implemented Swedish TTA criteria are safe, with levels of over- and undertriage remaining consistent after change of TTA criteria. The number of limited TTA diminished significantly with the new criteria, resulting in an increased efficiency in use of in-hospital resources, without compromising patient safety. Additional evaluation of TTA criteria are motivated in order to further reduce overtriage as well as for specific subgroups where undertriage is high, in particular elderly patients subject to fall injuries.

## Additional file

Additional file 1: **Table S1.** Definition of methods used for assessment of over- and undertriage. (DOCX 35 kb)
