# Supplementary material for: A prospective stepped wedge cohort evaluation of the new national trauma team activation criteria in Sweden – the TRAUMALERT study
Source: Scand J Trauma Resusc Emerg Med. 2019 Apr 30;27:52. doi: 10.1186/s13049-019-0619-1 (PMC6492485; doi:10.1186/s13049-019-0619-1)
Supplement: Supplementary file 1 — Table S1. Definition of methods used for assessment of over- and undertriage. (DOCX 35 kb) [file 13049_2019_619_MOESM1_ESM.docx]

Additional file 1: **Table S1** Definition of methods used for assessment of over- and undertriage

|  | Not severely injured, Injury Severity Score <15 | Severely injured, Injury Severity Score >15 | Sum |
| --- | --- | --- | --- |
| Full trauma team activation | A | B | A+B |
| Limited or no trauma team activation | D | E | D+E |
| Sum | A+D | B+E |  |
| Matrix method^7^ | Overtriage A/(A+B)*100 | Undertriage E/(D+E)*100 |  |
| Alternate method^17^ |  | Undertriage E/(B+E)*100 |  |
